# Prediction Is a Balancing Act: Importance of Sampling Methods to Balance Sensitivity and Specificity of Predictive Models Based on Imbalanced Chemical Data Sets

**DOI:** 10.3389/fchem.2018.00362

**Published:** 2018-08-28

**Authors:** Priyanka Banerjee, Frederic O. Dehnbostel, Robert Preissner

**Affiliations:** Structural Bioinformatics Group, Institute for Physiology, Charité – University Medicine Berlin, Berlin, Germany

**Keywords:** machine learning, DILI, sampling methods, Tox21, imbalanced data, molecular fingerprints, sensitivity-specificity balance, SMOTE

## Abstract

Increase in the number of new chemicals synthesized in past decades has resulted in constant growth in the development and application of computational models for prediction of activity as well as safety profiles of the chemicals. Most of the time, such computational models and its application must deal with imbalanced chemical data. It is indeed a challenge to construct a classifier using imbalanced data set. In this study, we analyzed and validated the importance of different sampling methods over non-sampling method, to achieve a well-balanced sensitivity and specificity of a machine learning model trained on imbalanced chemical data. Additionally, this study has achieved an accuracy of 93.00%, an AUC of 0.94, F1 measure of 0.90, sensitivity of 96.00% and specificity of 91.00% using SMOTE sampling and Random Forest classifier for the prediction of Drug Induced Liver Injury (DILI). Our results suggest that, irrespective of data set used, sampling methods can have major influence on reducing the gap between sensitivity and specificity of a model. This study demonstrates the efficacy of different sampling methods for class imbalanced problem using binary chemical data sets.

## Introduction

Increase in the number of new chemicals synthesis in past decades has resulted in constant growth in the development and application of computational models for prediction of activity as well as safety profiles of the chemicals (Mitchell, 2014; Banerjee et al., 2016; Hong et al., 2017). *In silico* models based on quantitative structure activity relationship modeling to molecular similarity based methods and machine learning models have been greatly successful in the field of computational drug design (Huang et al., 2016). Most of the time, computational models and their application must deal with imbalanced chemical datasets where one class is the majority class, outnumbers the other class in case of binary datasets. The minority class is oftentimes the class of interest. Constructing an accurate classifier from an imbalanced dataset is a challenging task. Traditional classifiers by maximizing the overall prediction accuracy tend to classify the data as majority class (Banerjee et al., 2016). Often the data comes from different sources like different experimental labs, experimental setup, as well as post processing of data can lead to increase in noise in the dataset. Highly imbalanced datasets are common in many pattern recognition tasks (López et al., 2013). For example, in medical datasets instances of diseased patients are typical rarer than instances of healthy individuals. Yet, it is the rare cases that attract the most interest, as identifying them enables the patient to be diagnosed and treated (Li et al., 2010). More precisely in chemical datasets, the problem of imbalanced data is common. Most of the *in silico* models for the prediction of bioactivities as well as toxicity profiles had to rely on an imbalanced dataset (Nanni et al., 2015). In binary classification, the underrepresented class is generally referred to as minority class, and the over represented class is referred to as majority. It is often observed that in case of an asymmetric class distribution, regular classifiers like support vector machine (SVM), as well as neural network (NN) tend to ignore the minority class, and treat them as noise resulting in a class boundary that unduly benefits the majority class (Maltarollo et al., 2015). Recently, many *in silico* models constructed on imbalanced data for prediction of chemical activity have achieved good performances in terms of accuracy and AUC (Drwal et al., 2015; Stefaniak, 2015; Capuzzi et al., 2016; Mayr et al., 2016; Banerjee and Preissner, 2018). However, very few have been able to handle the issues considering the false negatives and false positives. The imbalanced dataset is either dominated with positive instances or negative instances. Therefore, specificity and sensitivity of the model is highly important when addressing an imbalanced data set. Increase in sensitivity increases the true positive predictions of the model and reduces the false negatives. Similarly, improvement in the specificity increases the true negative predictions and hence reduces the false postives. Therefore, it is important that the gap between the specificity and sensitivity measures of a good model is as small as possible. In machine learning, many approaches have been developed to handle imbalanced data (Dubey et al., 2014; Beyan and Fisher, 2015; Pérez et al., 2016). The approach to handle imbalanced data can be in general classified into two broad categories as algorithmic or internal level and data or external level (López et al., 2013). On an internal level, there is the possibility of introducing a new design or tuning the existing one to handle the class imbalances (López et al., 2013). The major challenge with an internal approach is that they are specific to a classifier and the algorithm used for the classification task. On the external level, different types of data sampling methods are used such as under sampling and over sampling techniques. The external approach is straight forward, can be applied to any classifier, and at the same time incur the cost of over-fitting or losing the important information. This makes the external solution more adaptable and applicable.

In this study, we focus on different data sampling methods for improving the sensitivity as well as specificity of a classifier for prediction of compound activity. The study is based on two different data sets, coming from two different types of experimental data sources. The Tox21 dataset (Huang et al., 2016) and the Drug Induced Liver Injury (DILI) dataset (Chen et al., 2016; Thakkar et al., 2018). Furthermore, we introduce a new variant using maximum common feature (MCF) fingerprints in the sampling methods based on augmented random under and over sampling techniques. In this study, by using SMOTE (Synthetic Minority Over-Sampling Technique) over sampling method and Random Forest classifier, we have achieved a DILI prediction model with an accuracy of 93.00%, an AUC of 0.94, sensitivity of 96.00% and specificity of 91.00%. The experiments done in this study are based on two different data sets: four different prediction endpoints and multiple sampling methods using a uniform classifier are able to show the contribution of each sampling method on the predictive performance of the model. This study highlights the importance of reduction of gap between sensitivity and specificity in case of models trained on imbalanced chemical dataset.

## Materials and methods

### Data preparation

Four different types of imbalanced (having majority and minority class) data sets were used in this study. The data sets were standardized and curated as described in our previous work (Banerjee et al., 2016). The final datasets are reported in Table 1.

**Table 1:** The distribution of active and inactive class for both training and independent test set used in this study.

	**Prediction endpoints**	**Training set**	**Active/inactive**	**Independent test**	**Active/inactive ratio**
Tox21	AhR	6901	0.125	610	0.135
	ER-LBD	6801	0.053	600	0.034
	HSE	7328	0.043	610	0.039
NCTR	DILI	850	0.264	95	0.266

#### Tox21 dataset

The Toxicology in the twenty-first Century (Tox21) dataset used in this study is divided into three Tox21 assays (endpoints) such as (aryl hydrocarbon receptor (AhR), estrogen nuclear receptor alpha ligand-binding domain (ER-LBD) and heat shock protein beta-1 (HSE). All chemical structures were downloaded from the Tox21 Data Challenge 2014 website (https://tripod.nih.gov/tox21/challenge/index.jsp).

The Tox21 data consisted of the same number of datasets with three different cellular assays: NR-AhR, ER-LBD, and HSE as endpoints, as reported in our previous study (Banerjee et al., 2016). The Tox21 dataset can be used as a gold standard dataset for such comparative analysis, giving the standard experimental condition, the noise in the data is comparatively lower than in data coming from different sources as well as from different experimental setup.

#### Drug induced liver injury (DILI) dataset

The DILI dataset was prepared using different resources such as Liew dataset (Liew et al., 2011), Green and Xu dataset (Greene et al., 2010), DILIrank (Chen et al., 2016), and the Liver Toxicity Knowledge Base Benchmark Dataset (LTKB-BD) (Thakkar et al., 2018). The National Center for Toxicological Research (NCTR), U.S: FDA provides the benchmark dataset LTKB-BD. In this study, LTKB-BD dataset is used as the standard, and data from other sources were merged, keeping the activity/inactivity preference from LTKB-BD. The final dataset was then curated and standardized. The final DILI dataset contains 945 compounds (drugs).

### Molecular descriptors

To keep the analysis straight forward and more focused on the individual contribution of the sampling methods over non-sampling method; we used the MACCS fingerprints^1^ as it was reported as relatively better descriptor for prediction of Tox21 endpoints (Banerjee et al., 2016). MACCS fingerprints are designed on generic substructure keys. Additionally, the models were computed using Morgan fingerprints also known as circular fingerprints with radius 2 (Rogers and Hahn, 2010). The fingerprints were computed using the RDKit^2^ library in python.

### Sampling methods

Sampling techniques are widely used in the context of machine learning models to address the negative effect of an imbalanced training dataset. These external sampling methods are easy to implement and can be applied to any kind of classifier. Furthermore, depending on the individual classifier and possible algorithmic complexities, individual classifiers can be used to tune the model for better performance.

**Figure 1:**
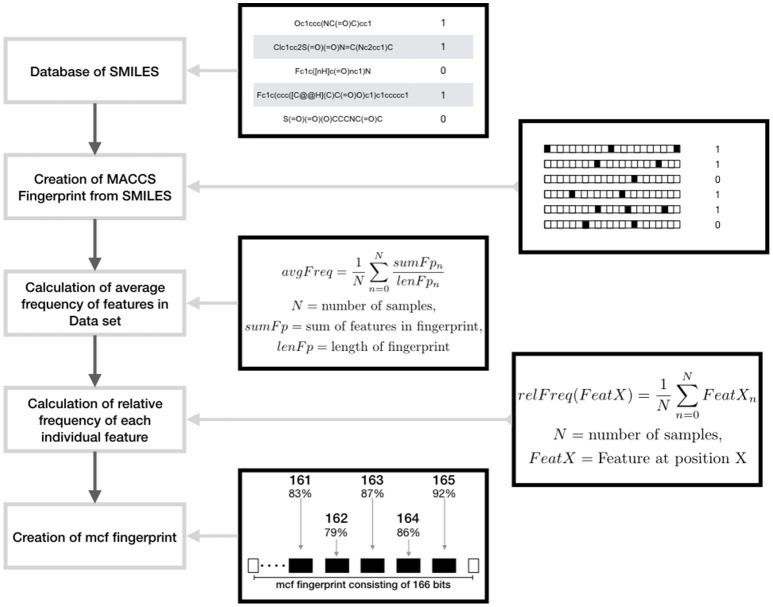
Schematic representation showing the design of maximum common feature (MCF) fingerprints using features derived from MACCS fingerprints.

The following are the different data sampling methods as used in this study:

- *No Sampling:* All the data were used without any manipulation, so called ‘original dataset’.
- Random Under Sampling (RandUS): The data points from the majority class are removed randomly.
- Augmented Random Under Sampling (AugRandomUS): Random under sampling in general removes instances of the dataset randomly. In this modified version, the randomness was reduced by utilizing a specifically calculated fingerprint called most common features (MCF) that incorporates all the common features in the data set. The features in this fingerprint are derived from MACCS fingerprints^1^ and Morgan fingerprints respectively. To produce this fingerprint the overall average frequency of all the features in the majority class is computed. Then, for each bit position of the fingerprint the relative frequency of ones in the complete data set is computed. If the relative frequency of a bit position is higher than the average frequency the respective bit position and the frequency is saved. Following the average number of features per fingerprint of the majority class is used to specify the number of the features per fingerprint of the MCF fingerprint, whereas the features themselves are specified by the saved features having the highest relative frequencies. Subsequently iteration is performed that is completed as soon as the majority data set is reduced to the size of the minority data set. In each step, a number of samples being the most similar to the MCF fingerprint are collected in a list. Then a number of instances is randomly chosen from the list and removed from the data set. Thereafter, a new MCF fingerprint is computed and the iteration is continued (Figure 1). In this way, the samples most similar to the MCF fingerprint are removed; the loss of variance of the majority set is decreased. In addition, the loss of information is reduced by removing a limited number of samples per calculated MCF fingerprints.
- *Random over sampling (RandOS):* Data points from the minority class are randomly chosen and added to the existing minority class.
- *Augmented Random Over Sampling (AugRandOS):* Random oversampling in this case also follows the same principle mentioned under the augmented random under sampling before. Only difference in this case, in each iteration step a list of samples most dissimilar to the MCF fingerprint is created. A part of the list is chosen randomly to be duplicated and added to the original data set. Since the samples most dissimilar to MCF are duplicated the loss of variance is relatively low. Both steps are repeated until the minority class consists of as many samples as the majority class.
- *K-Medoids Under Sampling (kMedoids1):* K-medoids is a clustering algorithm that is used to under sample the original majority class. A medoid is itself an instance of the majority class utilized as a cluster center that has the minimum average dissimilarity between itself and all majority data points in its cluster. The number of medoids is equal to the number of majority class instances. A sample is assigned to that cluster with which center it shares the highest similarity based on Tanimoto coefficient (Willett, 2003). For each of the medoids the sum of the similarities between itself and all samples belonging to its cluster is calculated. The algorithm tries to maximize the combination of these sums by performing iteration. The iteration is limited to 100 steps, in each of the iterations new medoids are randomly chosen and the overall sum of Tanimoto similarities is calculated. The set of medoids producing the highest sum is used as under sampled majority class. By means of clustering by similarity, this approach creates a subset of which each individual data point represents a group of structurally related molecules, in turn reducing the information lost by under sampling.
- *K-Medoids Under Sampling (kMedoids2):* Similarly to kMedoids1 this method starts with randomly choosing n samples as medoids, where n is equal to the number of data points in the minority class. For each of the chosen medoids, a total number of 30 iterations are assigned. In each iterative step, a medoid is exchanged with a random majority class sample, new clusters are computed and the cost is calculated using Tanimoto coefficient. The final set of medoids is chosen based on the maximum sum of similarities.
- *Synthetic Minority Over-Sampling Technique-using Tanimoto Coefficient (SMOTETC):* The SMOTE method creates synthetic samples of the minority class to balance the overall data set. Depending on the amount of oversampling a number of samples of the minority class are chosen. For each of those, the *k*-nearest neighbors are identified, utilizing the Tanimoto coefficient as similarity measure (Willett, 2003). The feature values of the new synthetic data points are set to the value occurring in the majority of the chosen sample and two of its *k*-nearest neighbors.
- *Synthetic Minority Over-Sampling Technique-using Value Difference Metric (SMOTEVDM):* This method is also based on SMOTE, but the *k-* nearest neighbors are chosen using the Value Difference Metric (VDM) as similarity measure. The VDM defines the distance between analogous feature values over all input feature vectors. More detailed information on the algorithm for computing VDM can be found here (Sugimura et al., 2008).

### Model construction

The Machine learning model was constructed using RF classifier. As reported in the earlier study RF model performed best (Banerjee et al., 2016). RF classifier is constructed from a multitude of decisions trees. In this study, we have used 1,000 estimators. The training set is divided into subsets and each of those is used to create a single classifier. A node is created by randomly choosing a number of features of the input vectors. The feature consisting of the most homogeneous binary split is then used as decision node. A majority vote of all trees is used to reach a prediction outcome. The advantage of using RF classifier is that it tends to avoid overfitting (Hansen et al., 2009; Flaxman et al., 2011; Díez-Pastor et al., 2015). The implementation of the model was done using the scikit-learn package for machine learning in python (Pedregosa et al., 2013).

### Model performance

For training the model, a 10-fold cross-validation was used, dividing the training dataset into 10 subsamples, keeping the distribution of the active and inactive class balanced. For each fold, 9 subsamples were used to train the model and the remaining 1 subsample as test set. The final model validation was computed using an independent test set. The performance strength for both the cross-validation and external validation using an independent test set, was measured using accuracy, the area under the curve (AUC) values of the receiver operating characteristic (ROC) curve, sensitivity, specificity, and f-measure (Banerjee and Preissner, 2018).

*Accuracy* of a model is defined as its ability to differentiate the actives and inactives cases correctly.

$$Accuracy\;=\frac{\Sigma \text{ True positives}+\;\Sigma \text{ True Negatives}}{\Sigma \text{ Positives}+\;\Sigma \text{ Negatives}}$$

*Sensitivity* describes the true positive rate i.e. the number of positive samples that were correctly identified as positive.

$$Sensitivity=\;\frac{\Sigma \text{ True Positives }}{\Sigma \text{ True Positives}+\;\Sigma \text{ False Positives}}$$

*Specificity* is the true negative rate i.e. the number of negative samples that were correctly identified as negative.

$$Specificity=\frac{\Sigma \text{ True Negatives}}{\Sigma \text{ True Negatives}+\;\Sigma \text{ False Positives}}$$

*F-measure* is the weighted average of precision and recall.

$$F-measure=\;{\text{2}}^{*}\frac{{\text{Precision}}^{*}\text{Recall}}{\text{Precision}+\text{Recall}}$$

*A receiver operating characteristic (ROC)-curve* is the plotting of the true positive rate against the false positive at various discrimination thresholds and is commonly used in binary classification. On the unit ROC space, a perfect prediction would yield an AUC of 1.0 and random results will be in points along with the diagonal with an AUC value of 0.5.

## Results

In this study four different data sets were used. The data sets contain binary imbalanced chemical data provided in Table 1. To keep the study uniform and comparable with our previous work (Banerjee et al., 2016), we have used MACCS molecular fingerprints and Morgan fingerprints, and Random Forest classifier for all the models. All the models and sampling methods were computed using python programming language and different machine learning packages. The results were obtained for both 10-fold cross validation and an independent test set for each of the prediction end points. We compared performance of the non-sampling based method with eight different types of sampling methods (see Methods).

### Tox21

**Figure 2:**
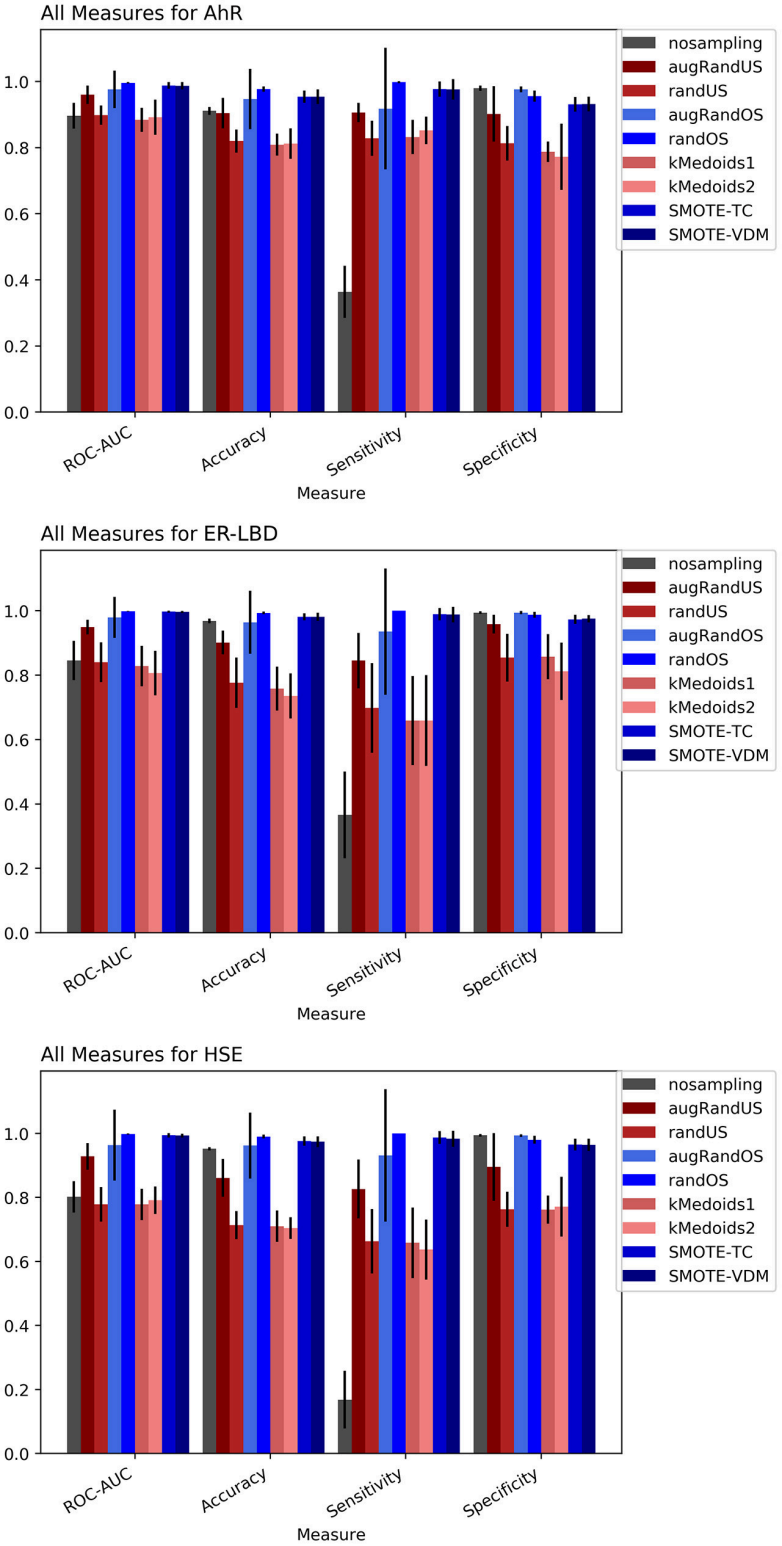
Performance measures for cross-validation -AhR **(A)**, ER-LBD **(B)**, and HSE **(C)** models based on Random Forest Classifier and MACCS fingerprints.

**Figure 3:**
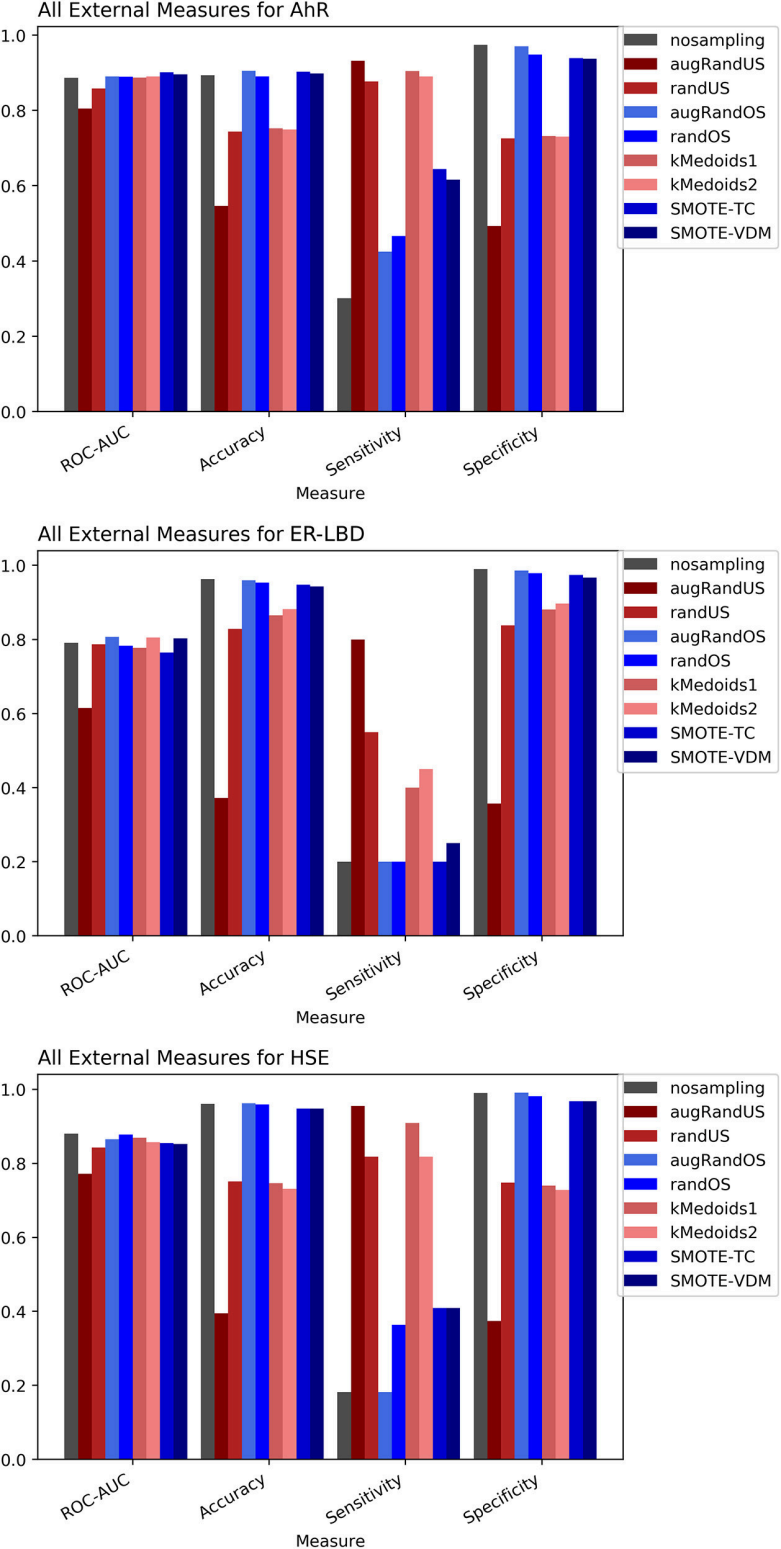
Performance measures for external validation -AhR **(A)**, ER-LBD **(B)**, and HSE **(C)** models based on Random Forest Classifier and MACCS fingerprints.

The Toxicology in the twenty-first Century (Tox21) dataset is divided into three Tox21 assays (endpoints) such as NR-AhR, ER-LBD, and HSE. All chemical structures were downloaded from the Tox21 Data Challenge 2014 website (https://tripod.nih.gov/tox21/challenge/index.jsp). Since the data is obtained from a standard experimental setup, it serves as a gold standard dataset for such analysis, assuming the experimental noise associated with the dataset is negligible. From the cross-validation results of all the three endpoints it is evident that non-sampling when compared to sampling methods performs equally well in terms of accuracy and AUC (Figures 2, 3). However, non-sampling method seems to perform poorly in terms of sensitivity and performs well in terms of specificity. This is because of the imbalanced data, the classifier tends to be biased toward the majority (negative) class, a problem non-sampled data cannot address. This gives rise to the question of false positives and false negatives. On the other hand, different sampling methods seem to handle the issue of sensitivity (true positives) without compromising on the other performance measures such as accuracy, AUC and specificity. Sampling methods used in this study like augmented random oversampling that incorporates most common feature (MCF) fingerprints perform equally well in terms of all the measures for all the three end points (Figures 2, 3). However, the standard deviation of sensitivity is high in these sampling methods. Similarly, SMOTE-TC and SMOTE-VDM achieved good performance in all the parameters. Since, the data set is dominated by negative instances; it is expected to have low sensitivity and high specificity. The challenge was to increase the sensitivity without compromising on the accuracy, AUC and specificity. The external validation results of the Tox21 dataset for NR-AhR suggests SMOTE-TC sampling method reached superior performance compared to non-sampling and other sampling methods (Figure 3). Similarly, this is true for SR-HSE end points. In case of ER-LBD, the observation is opposite. Most of the under sampling techniques including the k-medoids methods perform well in terms of sensitivity; however there is a sharp decrease in specificity (Supplementary Figures 1–4). The over sampling methods like SMOTE-TC and SMOTE-VDM perform equal to the non-sampling method in terms of accuracy, AUC and specificity. However, the sensitivity of this model is low. This could be because the chemical space of ER-LBD is highly conserved as we have seen in our previous study (Banerjee et al., 2016). Therefore, increasing the number of instances in this dataset by over sampling does not help, as the data is homogenous and rare events are not captured completely. It is also observed that the positives in the external set are highly diverse or similar to the negative class instances when compared to the training set. Furthermore, F1 measure values are higher for the sampling methods for all the three prediction endpoints compared to non-sampling method (provided as Supplementary Figure 5). Similar performance can be observed in case of Morgan fingerprints, sampling methods performed better compared to non-sampling methods with respective to balance between specificity and sensitivity and F1 measure (see Supplementary Figure 6) in the cross-validation. In case of classifiers trained on real datasets, it is often observed that where actives (minority class examples) in the training sets and test sets are very different. As a result, the internal cross-validation score might be higher compared to that of the external scores as seen in case of F1 measures for the ER-LBD model (Supplementary Figure 5). In such cases using adversarial cross-validation or CLUSTER cross-validation can provide an interesting solution (Mayr et al., 2016; Banerjee et al., 2018). Comparison between the performances of the models based on MACCS and Morgan fingerprints are provided as Supplementary Figures 8–10 for AR-AhR, ER-LBD, and HSE respectively.

### DILI

The DILI dataset was obtained from sources like DILIrank (Chen et al., 2016), and the NIH LiverTox database (Thakkar et al., 2018) and were normalized and curated to be used in this study as explained in our previous work (Drwal et al., 2015). It is well known that the mechanisms of DILI are not only complicated but at the same time diverse. This makes it even more challenging to produce an optimal prediction. However, the DILI chemical space contains certain common substructures that were used to train the model. In this case, the dataset was dominated by negative class samples making it imbalanced. Hence it is interesting to validate if the sampling methods can be helpful.

**Figure 4:**
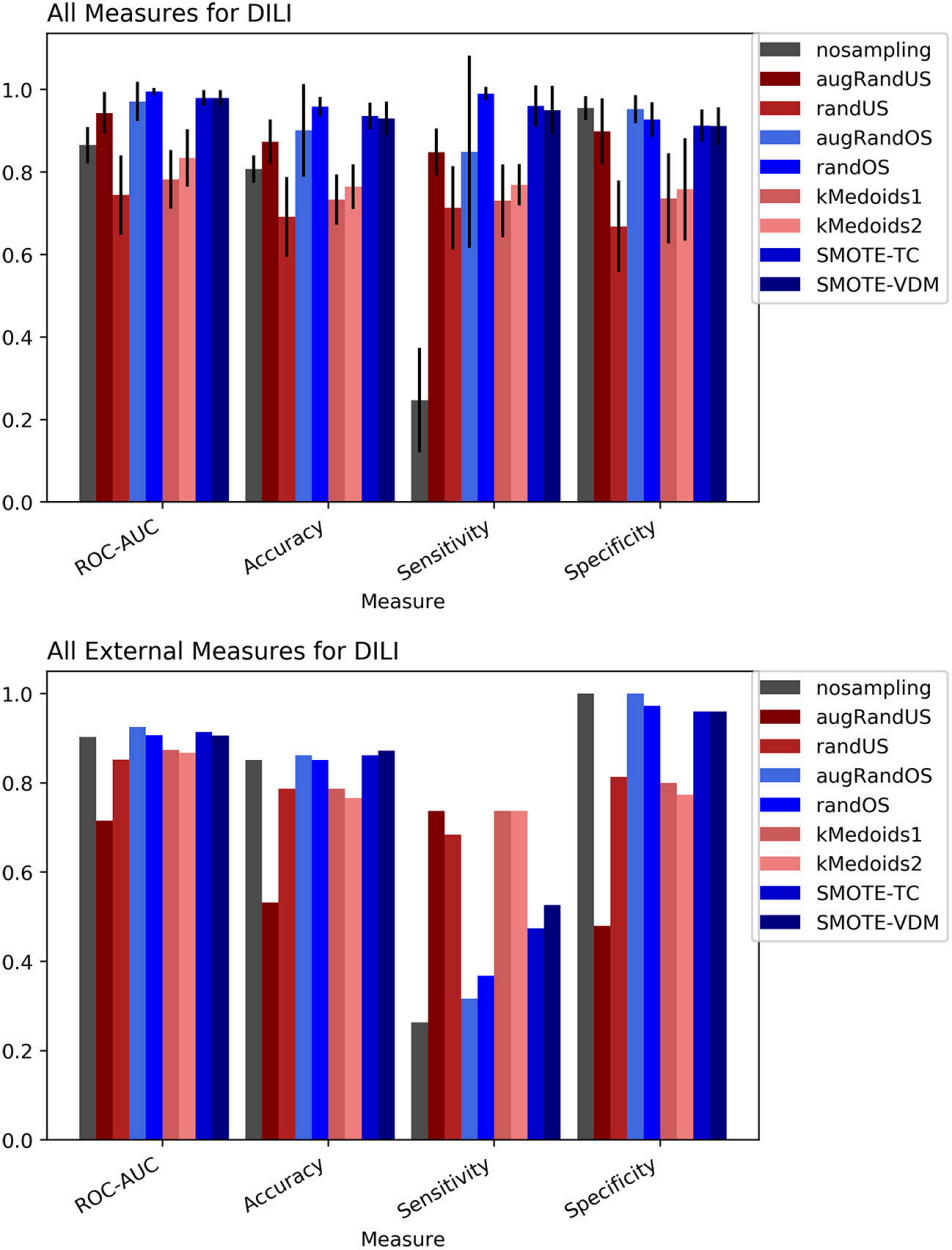
Performance measures for cross-validation and external validation for DILI model based on Random Forest Classifier and MACCS fingerprints.

To evaluate the performance, 10-fold cross-validation was performed using the different sampling methods and non-sampling method. From the cross-validation results it is evident that the SMOTE-TC over sampling method outperforms other sampling methods as well as the non-sampling method. Using, SMOTE-TC an accuracy of 93.5%, an AUC of 0.94, sensitivity of 96.00% and specificity of 91.00% has been achieved on our dataset (Figure 4). The standard deviation of all the measures using SMOTE-TC is low when compared to other sampling methods. The non-sampling method performs poorly in terms of sensitivity and the standard deviation is high. This holds true for both MACCS and Morgan fingerprints based respective models. However, it is observed that MACCS keys performed slightly better than the Morgan fingerprints (Supplementary Figure 6). A detailed comparison between the performances of the models based on MACCS and Morgan fingerprints are provided as Supplementary Figure 7.

The external validation results of the DILI dataset show that overall all the sampling methods have achieved greater balanced between sensitivity and specificity measures than the non-sampling method.

However, as expected under sampling methods increased the sensitivity of the model while decreasing the specificity compared to the original dataset. In contrast, over sampling resulted in higher specificity but lower sensitivity than under sampling approaches. Overall, the SMOTE and k-medoids methods performed optimally good achieving accuracy above 80.00%, an AUC above 0.90 and sensitivity and specificity above 90.00%. The k-medoids methods performed extremely well on reducing the gap between sensitivity and specificity (Figure 4). It is also observed that the F-measure for both the cross-validation and external validation is higher in case of sampling methods compare to non-sampling method (see Supplementary Figure 5).

## Discussion

**Figure 5:**
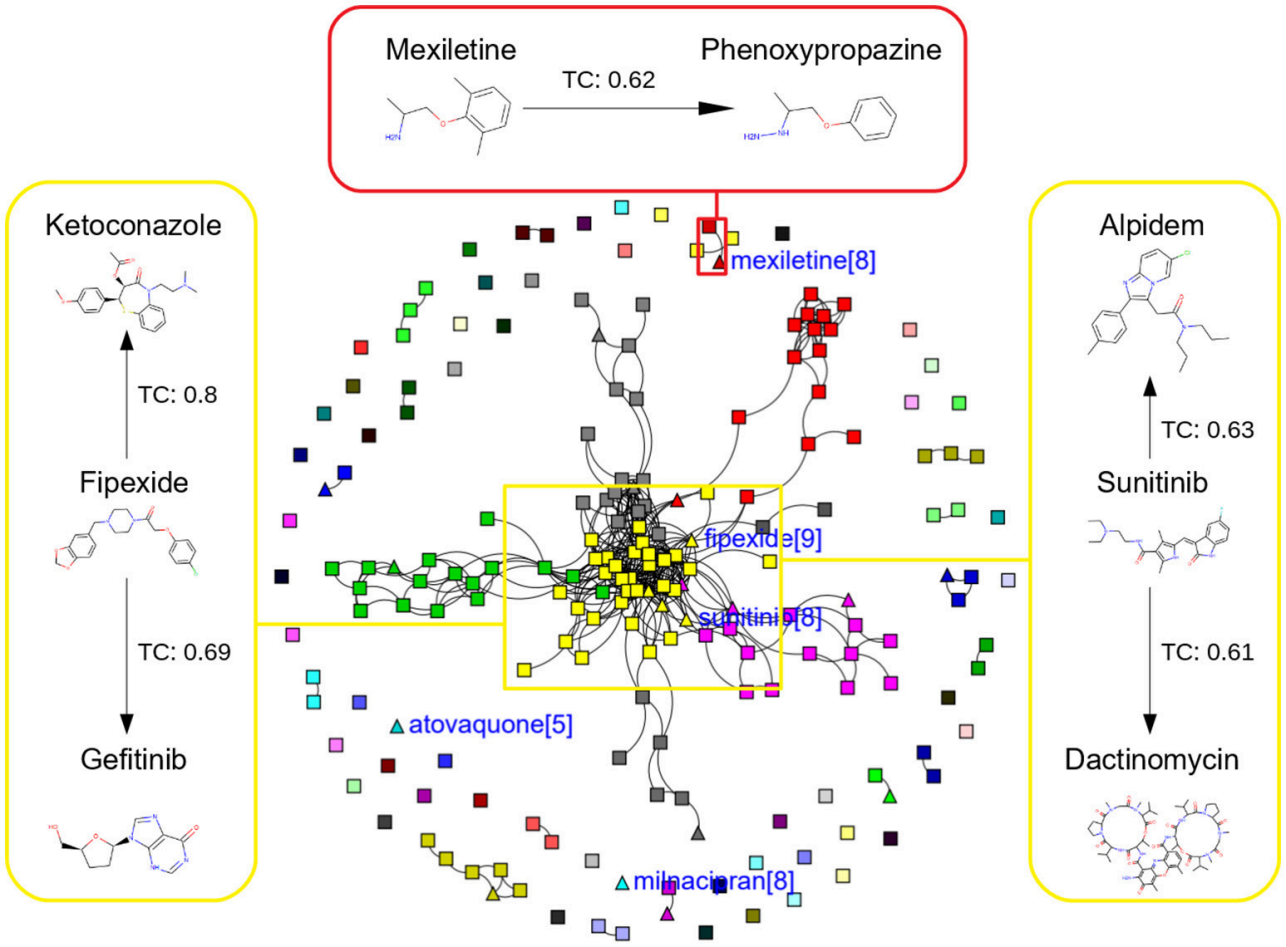
Chemical Space Networks of the actives of external test set (triangle) and actives of training set (square). The CSNs reveals that mexiletine (external test active) compound having similarity with phenoxypropazine (training set active) is incorrectly predicted by all the sampling methods as inactive. Similarily fipexide, sunitinib (external set active) are incorrectly predicted by all the sampling methods.

**Figure 6:**
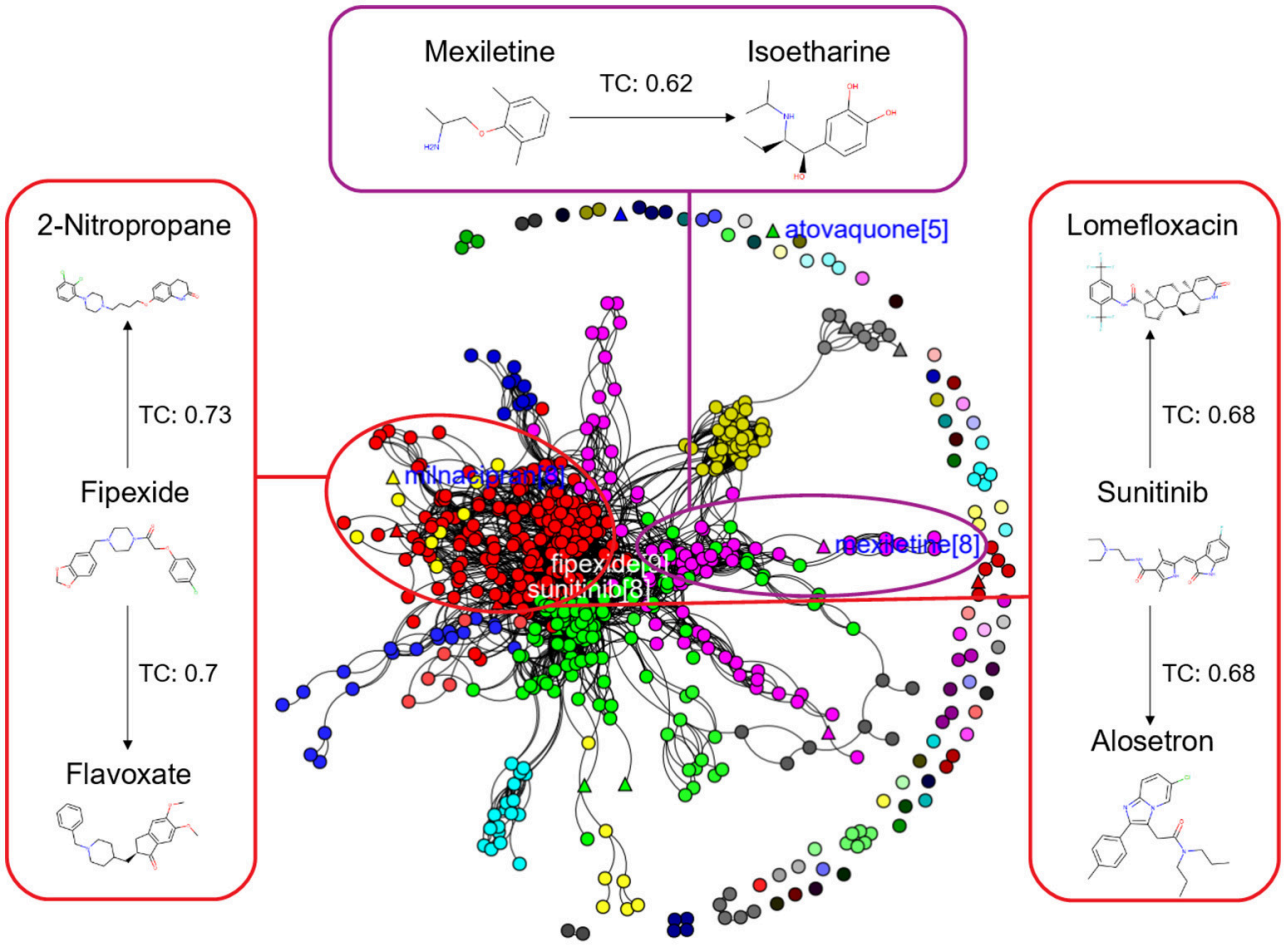
Chemical Space Networks of the actives of external test set (triangle) and inactives of training set (circle). The CSNs reveals that mexiletine (external test active) compound having similarity with isoetharine (training set inactive) is incorrectly predicted by all the sampling methods as inactive (false negative). Similarily fipexide, sunitinib (external set actives) are incorrectly predicted as inactive by all the sampling methods.

In this study, we present four different models based on Random Forest classifier and eight different sampling methods. Three models are based on Tox21 dataset and with respective endpoints (NR-AhR, NR-ER-LBD, and SR-HSE). The fourth model is trained on LiverTox database (see Methods) for the prediction of DILI. All the models are trained using MACCS molecular fingerprints as descriptors. One of the major outcomes of this study is the improvement of the sensitivity of all the models using over sampling methods like SMOTE and under sampling methods like k-medoids techniques without compromising on the accuracy, AUC and specificity of the models. Secondly, the DILI model proposed in this study achieved an accuracy of 93.00%, an AUC of 0.94, sensitivity of 96.00% and specificity of 91.00% on the cross-validation and an accuracy of 83.00%, AUC of 0.89, sensitivity of 84.20% and specificity of 82.70% on the independent set, using SMOTE-TC over sampling method. Additionally, in this study a newly designed most common features (MCF) fingerprint representation of the active space in the respective training sets, are used to modify random under sampling and over sampling techniques. The classifier and the descriptor in this study, has been kept uniform to measure the individual contribution of the sampling methods. The study is further compared to our previous work (Banerjee et al., 2016) and the current study outperforms in terms of sensitivity. The DILI model proposed in this study, showed better performance when compared to other models published for DILI predictions (Table 2). When compared with a well performing model for DILI prediction based on deep learning (Xu et al., 2015), it is observed in this study that an external tuning of data by sampling methods can produce equally good performance even using computationally less expensive algorithms like RF. The introduction of MCF fingerprints in both augmented random over sampling and augmented random under sampling methods, resulted in increase in both sensitivity and specificity of all the models, without loss of performance in accuracy and AUC-ROC values in the cross-validation set. However, the same was not observed on the external validation sets. This approach resulted in reduced sensitivity and specificity on external sets for the ER-LBD model. It is worth mentioning that the performance of each of the sampling methods is highly depended on the chemical space of the respective models. There is no clear winner among the eight sampling methods. However, in general it is seen that sampling is a sensible way to reduce the gap between sensitivity and specificity of a model. K-medoids sampling tends to show similar behavior for all the models, both on cross-validation and external validation sets. Thus, reflecting that performance of sampling methods could be highly dependent on the chemical space of the data of respective models. To understand the strength of individual sampling methods as well as the influence of distribution of chemical data in model training, we analyzed the Chemical Space Networks (CSNs) for all the models (Maggiora and Bajorath, 2014). Due to relatively large number of compounds present in the Tox21 datasets, it is difficult to visualize network and its interpretability. Hence, it was more prudent to visualize, display and analyze a moderately size compound data set such as DILI. In CSNs, compounds are represented as nodes, and the shape of the nodes in this study represents the activity of the compound and the color represents the compound cluster. The edges connecting the nodes represent the pairwise similarity relationships. For each dataset two CSNs were produced. The first, containing the actives of the test set and training set, and the second comprising of the actives of the test set and inactives of the training set. This was done to analyse the compound distributions and to assess the diversity of the compound in both training and test data sets as well as to visualize the active borderline cases. The CSNs were designed by clustering the actives of the test set and actives of the training set (Figure 5) as well as the actives of the test set and inactives of the training set (Figure 6) using structutal similarity. It can be seen that the active compounds of the training set are structurally more diverse and produce more singletons compared to the inactives, which exist in comparatively larger clusters. Since, molecular mechanism behind drugs induced liver injury is a highly complicated and diverse phenomenon (Liyun and Neil, 2013), the drugs which are found to be active for DILI; makes the DILI relevant chemical space very diverse. On the other hand, it reflects the model tend to learn the rules based on the most of negative instances of the training data, and hence results in better predictions of true negatives compared to true positives. This clearly demonstrate why in case of non-sampled data, the minority class predictions tends to have worse performance than the majority class predictions, and the minority class predictions are misclassified much more frequently (14 out of 20 actives compounds) than majority class examples (not a single majority class compound misclassified) (Table 3). Thus, it is noticed that when learning from data sets with a high degree of class imbalance, classifiers rarely predict the minority class (Provost and Weiss, 2011). Therefore, introducing sampling methods like augmented random under sampling using MCF fingerprints and *KMedoids2* improved the predictions of the minority class with a slight decrease in the majority class predictions (Table 3). Overall it can be said that sampling methods helps to overcome biased behavior and complexities when training positive instances are costly.

**Table 2:** Comparison of the DILI model presented in this study with other published DILI models.

**Models**	**Accuracy (%)**	**Sensitivity (%)**	**Specificity (%)**	**AUC-ROC**
DILI (this study) Random Forest and SMOTE -TC sampling method	93.00	96.00	91.00	0.94
(Xu et al., 2015) Deep learning	86.90	82.50	92.90	0.95
(Zhang et al., 2016)(Zhang et al., 2016) Pattern recognition method	66.00	85.00	34.00	0.55

**Table 3:** Total number of false positives and false negative predicted by different non/sampling methods for DILI model based on Random Forest classifier and MACCS fingerprints.

**Active compounds External Test set**	**No sampling**	**AugRandUS**	**randUS**	**AugRandOS**	**randOS**	***kMedoids1***	***KMedoids2***	***SMOTETC***	***SMOTEVDM***
Etravirine	X	^*^	^*^	^*^	^*^	^*^	^*^	^*^	^*^
Levofloxacin	X	X	^*^	X	X	X	^*^	^*^	^*^
Ciprofloxacin	X	X	X	X	X	X	^*^	X	X
Clozapine	X	^*^	^*^	X	X	^*^	^*^	X	^*^
Milnacipram	X	^*^	X	X	X	X	X	X	X
Nefazodone	X	^*^	^*^	X	X	^*^	^*^	^*^	^*^
Mexiletine	X	^*^	X	X	X	X	X	X	X
Sunitinib	X	X	X	X	X	^*^	X	X	X
Pazopanib	X	^*^	^*^	X	X	^*^	^*^	X	X
Alpidem	X	X	X	X	X	^*^	^*^	X	X
Fipexide	X	X	X	X	X	X	X	X	X
Exifone	X	^*^	^*^	X	^*^	^*^	^*^	^*^	^*^
Atovaquone	X	^*^	^*^	X	X	^*^	^*^	X	X
Trimethadione	X	^*^	^*^	X	X	^*^	^*^	X	X
No of false negatives	14/20	5/20	6/20	13/20	12/20	5/20	4/20	10/20	9/20
No of false positives	0/75	39/75	14/75	0/75	2/75	15/75	17/75	3/75	3/75

^X~^actives misclassified by model trained with respective sampled dataset.

* *~ actives correctly classified by model trained with respective sampled dataset*.

Additionally, it is noticed that in the case of an active compound in the test set sharing equally high structural similarities with active and inactive compounds of the training set, the model fails to classify it to the right class. Such is the case of Fipexide, which is an active DILI compound, but was wrongly predicted by 8 different sampling methods. This is because Fipexide shares strong similarities with active compounds (ketaconazole and Gefitinib) as well as inactive compounds (2-Nitropropane and Flavoxate). Similarly this can be observed in the case with Maxiletine, Sunitinib and others shown in the Figures 5, 6. These borderline and noisy minority class samples in the test set are placed close to the complex, decision boundary between the classes. Thus, they are misclassified by similar neighbors from the opposite class located on the other side of the boundary. It can be said that these data points can often be outliers, which represents a rare but valid events. Therefore, they need to be handled specially, or by re-labeling of the majority class (negative) class instances.

Often, it is observed the specificity and sensitivity of a classification with class imbalance problem is inversely related. Selecting the optimal balance between the sensitivity and specificity of a classifier is entirely dependent on the goal of the classification task. Generally, an *in silico* screening method such as DILI predictions should be highly sensitive, whereas follow-up confirmatory methods or experimental tests should be highly specific. Certainly, this also opens scope and needs for further updates and training of the models; whenever new data instances are available. Thus, justifying prediction is indeed a balancing act between sensitivity and specificity which needs a continuous introspection.

## Conclusions

Constructing an accurate classifier from an imbalanced chemical dataset is indeed a challenging task. Because traditional classifiers tend to maximize the overall prediction accuracy, they become biased toward the majority class. Given a particular prediction task on imbalanced data, one relevant question to ask is that which type of sampling methods should be used? Though a large number of sampling methods are available for addressing the data imbalance problem, as it is typically the case, there is no single sampling method which works best for all problems. The choice of the data sampling methods greatly depends on the nature of the dataset and the primary learning goal. Our results suggest that, irrespective of data sets used, sampling methods can have major influence on the gap between sensitivity and specificity of a trained model over non-sampling method. This study demonstrates the efficacy of different sampling methods for class imbalanced problem using binary chemical data sets.

Furthermore, it is important to state that even though sampling methods performs better compared to non-sampling method. However, unfortunately, there is no single method that can work well for all problems. Such is in the case of ER-LBD model, though sampling methods achieved overall better scores than non-sampling method in terms of all the performance measures. However, the overall F1 measures of the external set for all the models were poor compared to superior scores of the cross-validation set. It will be wise to also state that, F1 measure is a combined metric, so it is advisable to calculate precision and recall separately see Supplementary Figure 11, and make a decision based on the goal in mind. In case of binary dataset, there is always a trade-off between precision and recall. If one chooses to optimize precision of the model, disfavoring recall and vice-versa, this will result in the dropping of the harmonic mean. Ideally, it will be great to have both precision and recall as equal, making the task often challenging. However, using different cross-validation techniques like adversarial cross-validation, more discriminative features, better algorithm or combination of different algorithm, using higher weights to the minority class besides using sampling methods can further improve the model performance.

In this study, we analyzed and validated the importance of different sampling methods over non-sampling method, to achieve a well-balanced sensitivity and specificity of a machine learning model trained on imbalanced chemical data. Different from earlier studies, our calculations based on sampling methods have stressed the importance of considering different sampling methods when training a Random Forest classifier using imbalanced chemical data sets. Here, we have used two different datasets and four different endpoints, the “Toxicology in the twenty-first Century' dataset and the Drug Induced Liver Injury dataset from NCTR. Additionally, our study has achieved an accuracy of 93.00%, an AUC of 0.94, F1-measure of 0.90, sensitivity of 96.00% and specificity of 91.00% using SMOTE sampling and Random Forest classifier for the prediction of Drug Induced Liver Injury (DILI).

The DILI model presented in this study aims to facilitate the DILI risk prediction in humans and will be made freely available via ProTox-II, computational toxicity prediction platform (http://tox.charite.de/protox_II). Training and test sets and scripts generated and/or analysed in the current study are available from the corresponding author upon request.

## Author contributions

PB and RP conceived the idea. PB designed the workflow and curated the dataset. PB and FD developed the models, performed the sampling calculations and validations of the models. PB and FD wrote the manuscript. PB, FD, and RP proofread and revised the manuscript.

### Conflict of interest statement

The authors declare that the research was conducted in the absence of any commercial or financial relationships that could be construed as a potential conflict of interest.

## Footnotes

^1^*MACCS Structural Keys*. San Diego, CA: Accelrys. Available online at: http://accelrys.com.

^2^*RDKit: Open-Source Cheminformatics Software*. Available online at: http://www.rdkit.org

## Abbreviations

AhR: aryl hydrocarbon receptor
ER-LBD: estrogen receptor ligand binding domain
HSE: heat-shock element
NB: Naive Bayes classifier
AUC: area under the curve
RF: random forest
TC: Tanimoto Coefficient
Tox21: Toxicology in the twenty-first century
DILI: Drug Induced liver Injury
SMOTE: Synthetic Minority Over-Sampling Technique.
