# Supplementary material for: Prediction Is a Balancing Act: Importance of Sampling Methods to Balance Sensitivity and Specificity of Predictive Models Based on Imbalanced Chemical Data Sets
Source: Front Chem. 2018 Aug 28;6:362. doi: 10.3389/fchem.2018.00362 (PMC6149243; doi:10.3389/fchem.2018.00362)

Supplementary Material

Prediction is a Balancing Act: Importance of Sampling Methods to Balance Sensitivity and Specificity of Predictive models based on Imbalanced Chemical datasets

Priyanka Banerjee* , Frederic O. Dehnbostel , Robert Preissner

*** Correspondence:** Corresponding Author: priyanka.banerjee@charite.de

1.1 Supplementary Figures

SIFigure1: Accuracy measures for cross-validation (internal) and external validation of -AhR (a), ER-LBD (b) and HSE (c) and DILI (d) models using MACCS fingerprints.

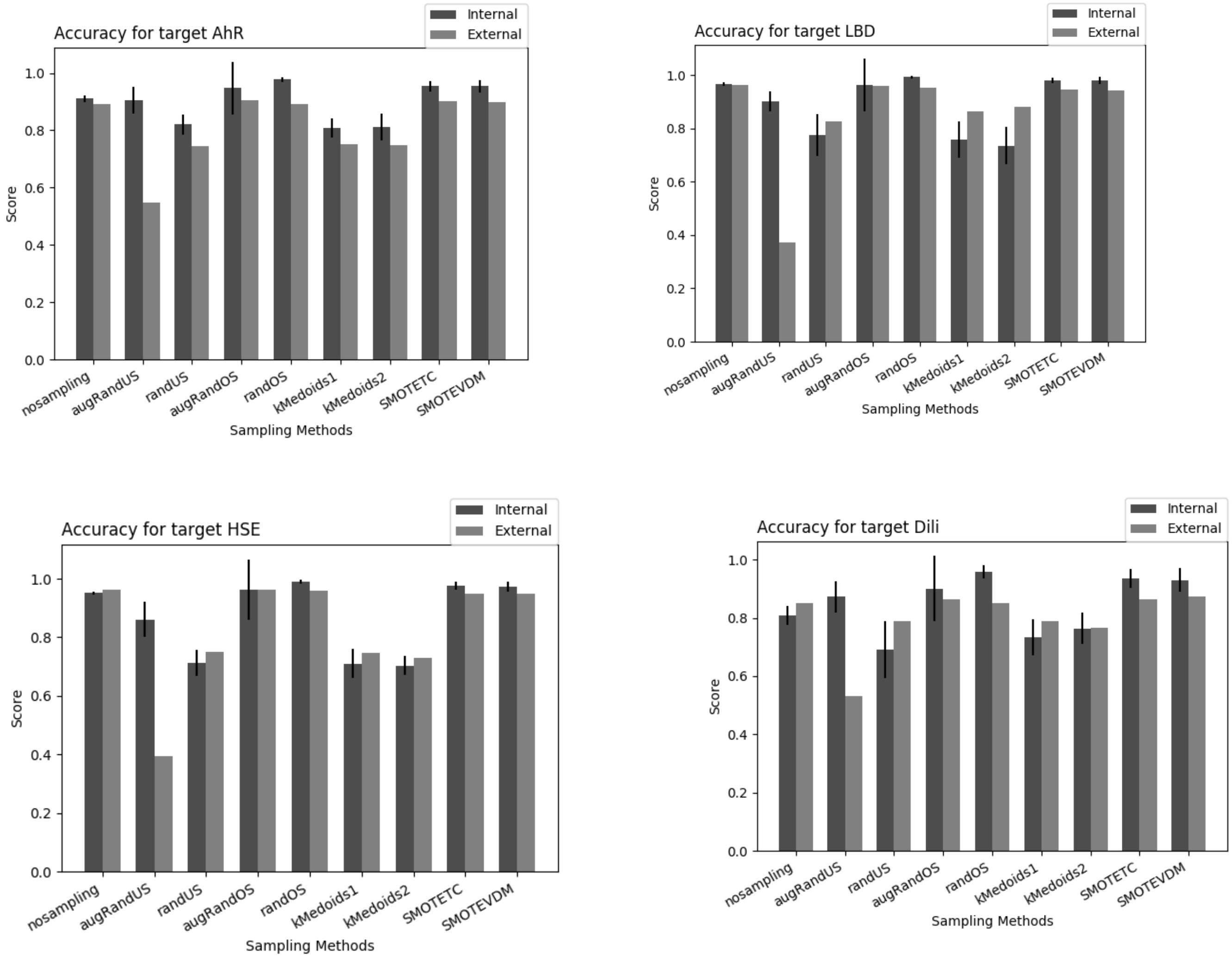

SIFigure2: AUC ROC measures for cross-validation (internal) and external validation of -AhR (a), ER-LBD (b) and HSE (c) and DILI (d) models using MACCS fingerprints.

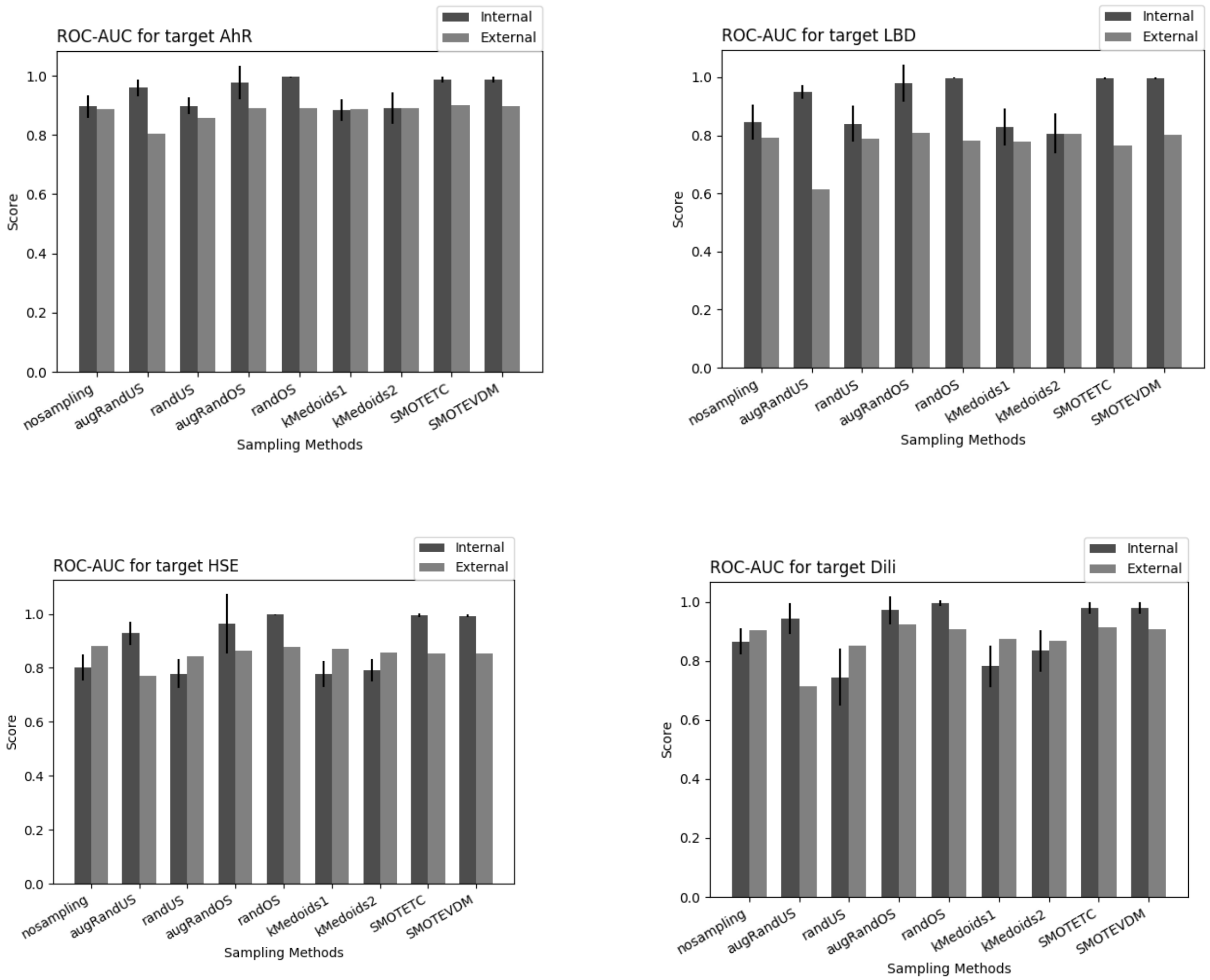

SIFigure3: Sensitivity measures for cross-validation (internal) and external validation of -AhR (a), ER-LBD (b) and HSE (c) and DILI (d) models using MACCS fingerprint.

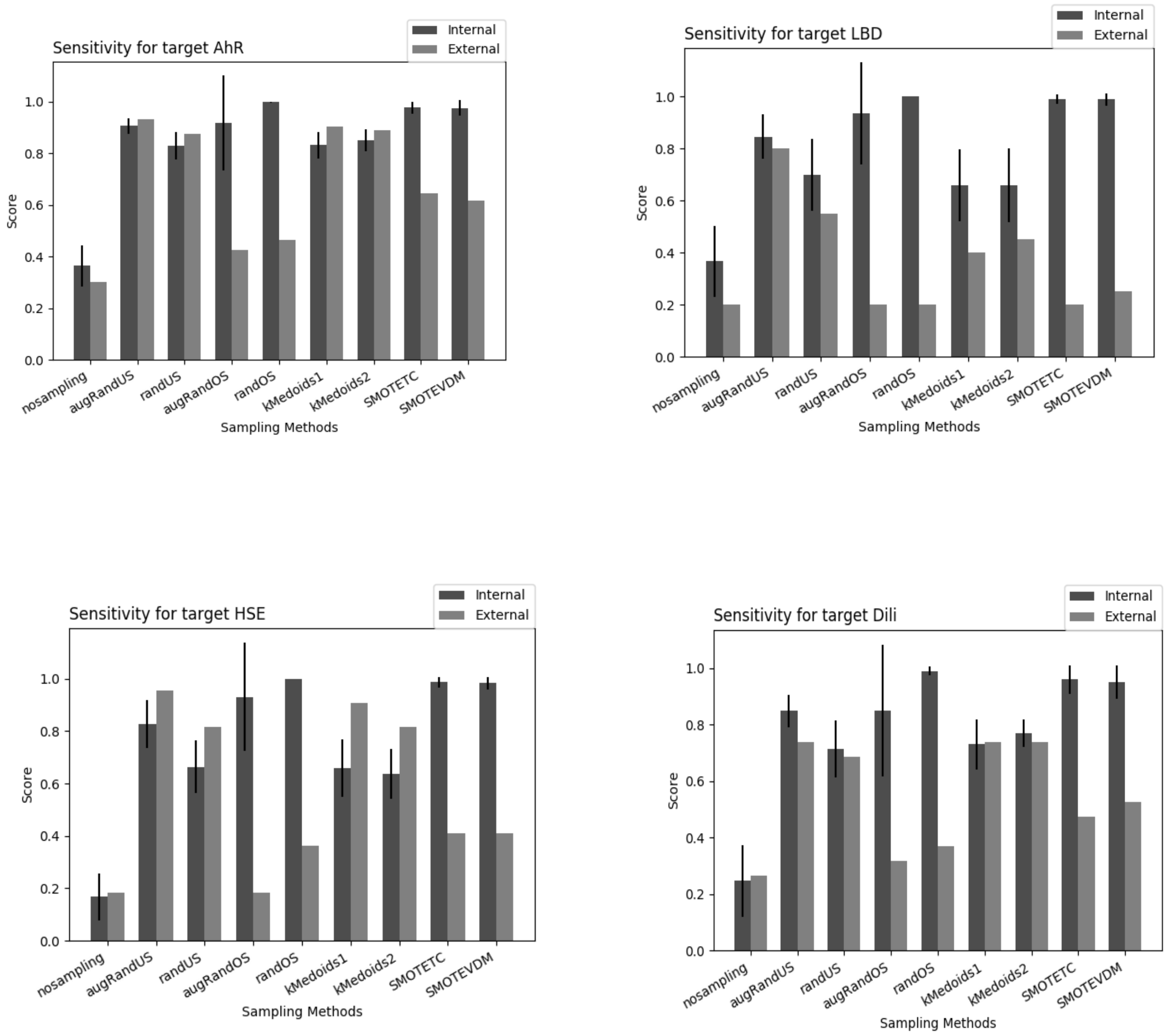

SIFigure4: Specificity measures for cross-validation (internal) and external validation of -AhR (a), ER-LBD (b) and HSE (c) and DILI (d) models using MACCS fingerprint.

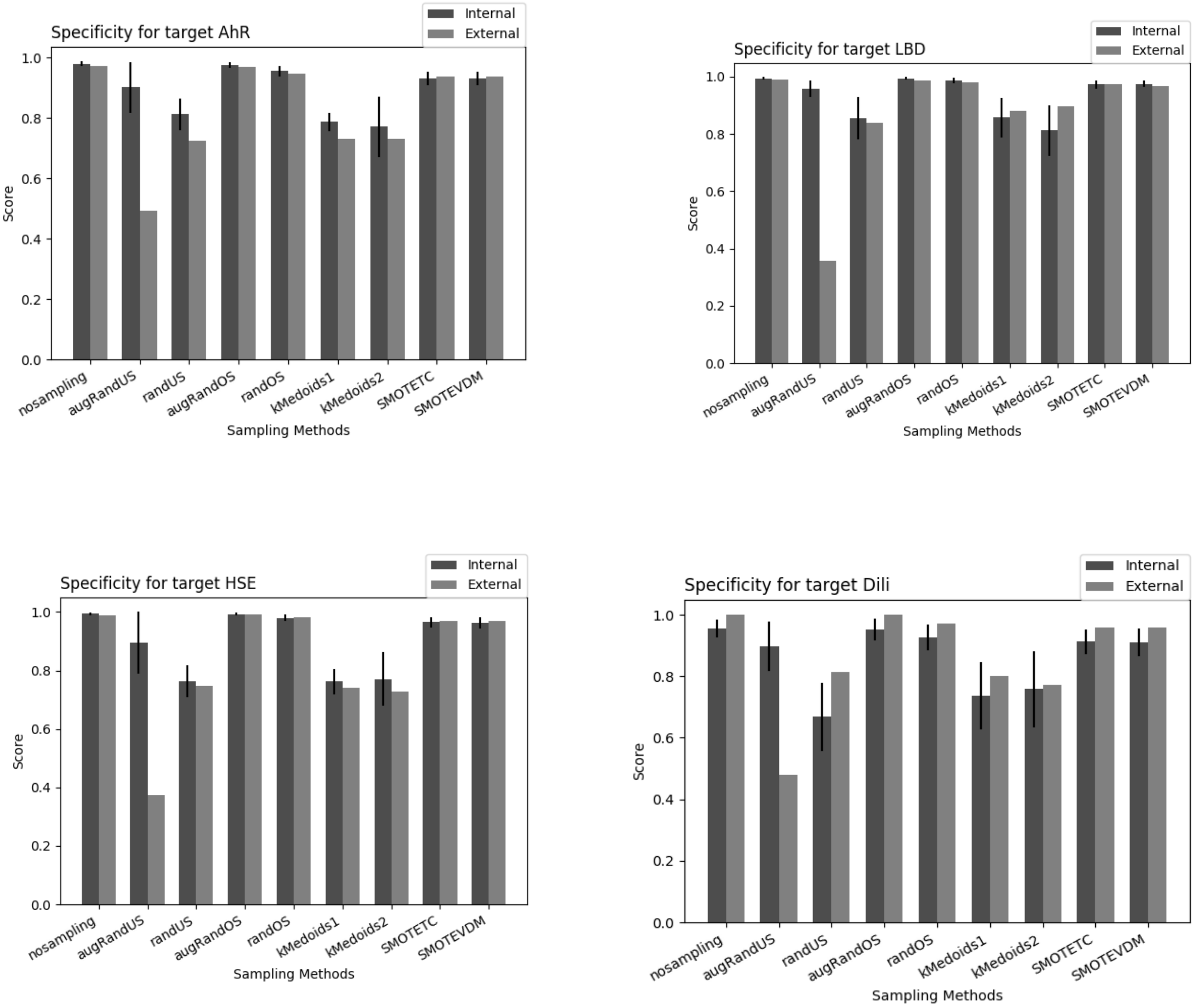

SIFigure5: F1 measures for cross-validation (internal) and external validation of -AhR (a), ER-LBD (b) and HSE (c) and DILI (d) models using MACCS fingerprint

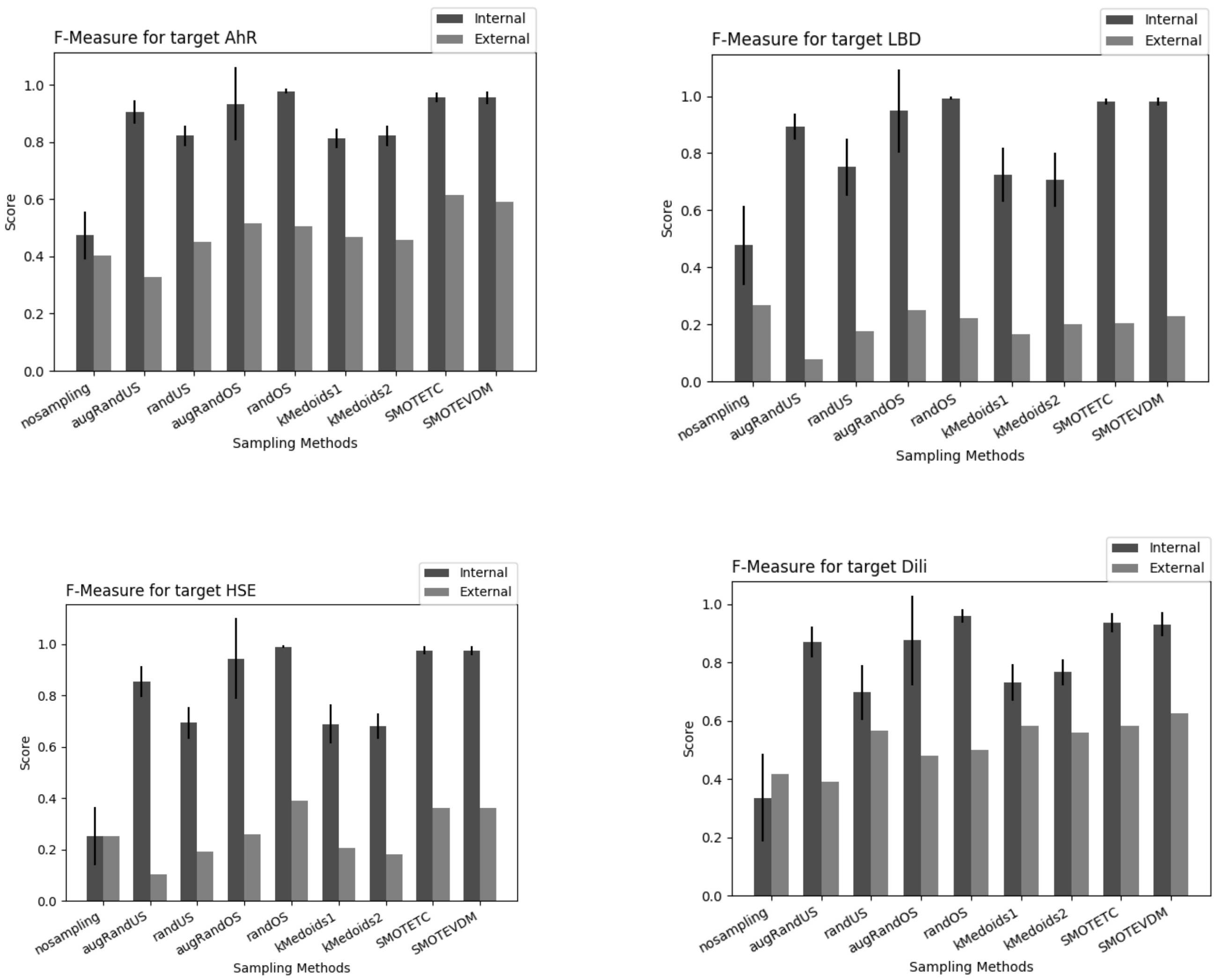

SIFigure6: All performance measures for cross-validation and external validation of -AhR (a), ER-LBD (b) and HSE (c) and DILI (d) models using Morgan fingerprint.

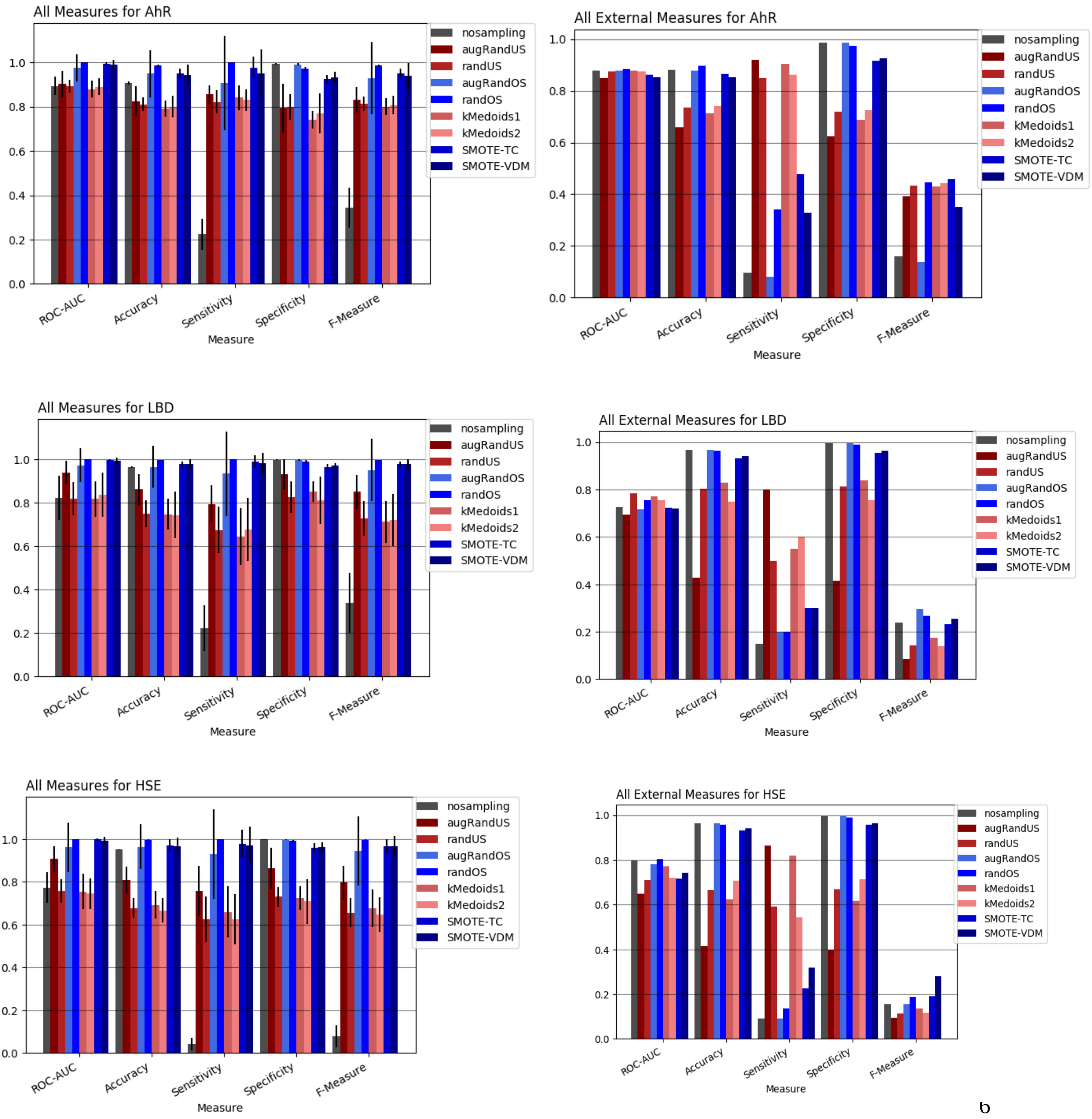

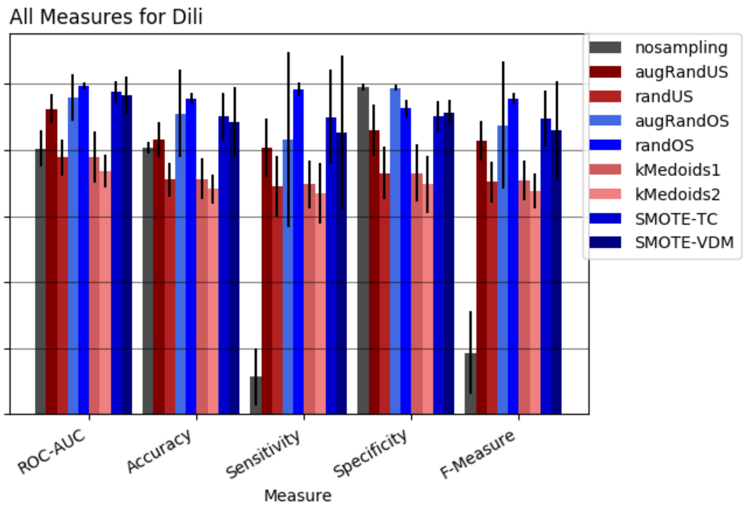
All Measures for Dili
nosampling
augRandUS
randUS
augRandOS
randOS
kMedoids1
kMedoids2
SMOTE-TC
SMOTE-VDM
ROC-AUC
Accuracy
Sensitivity
Specificity
F-Measure
Measure

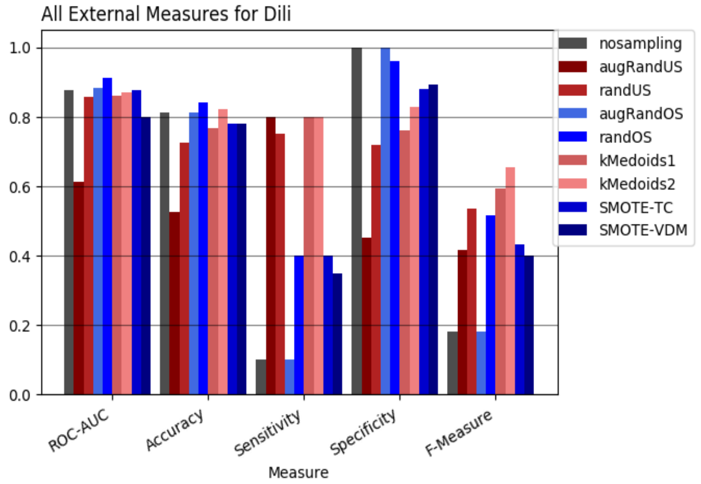
All External Measures for Dili
nosampling
augRandUS
randUS
augRandOS
randOS
kMedoids1
kMedoids2
SMOTE-TC
SMOTE-VDM
ROC-AUC
Accuracy
Sensitivity
Specificity
F-Measure
Measure

SIFigure7: Comparison between DILI models based on MACCS and Morgan fingerprints with respect to of all performance measures for cross-validation (internal) and external validation.

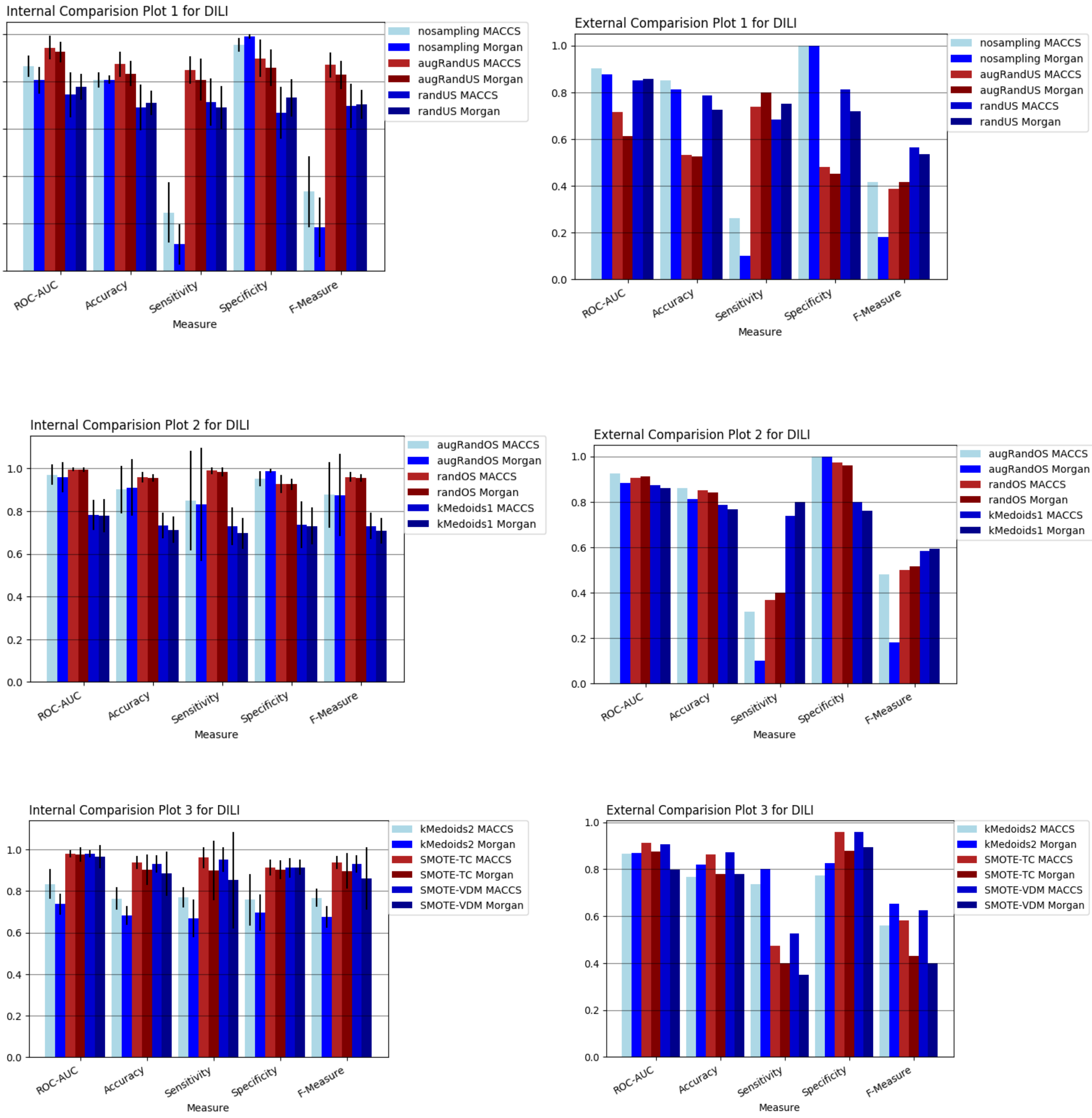

SIFigure8: Comparison between AhR models based on MACCS and Morgan fingerprints with respect to of all performance measures for cross-validation (internal) and external validation.

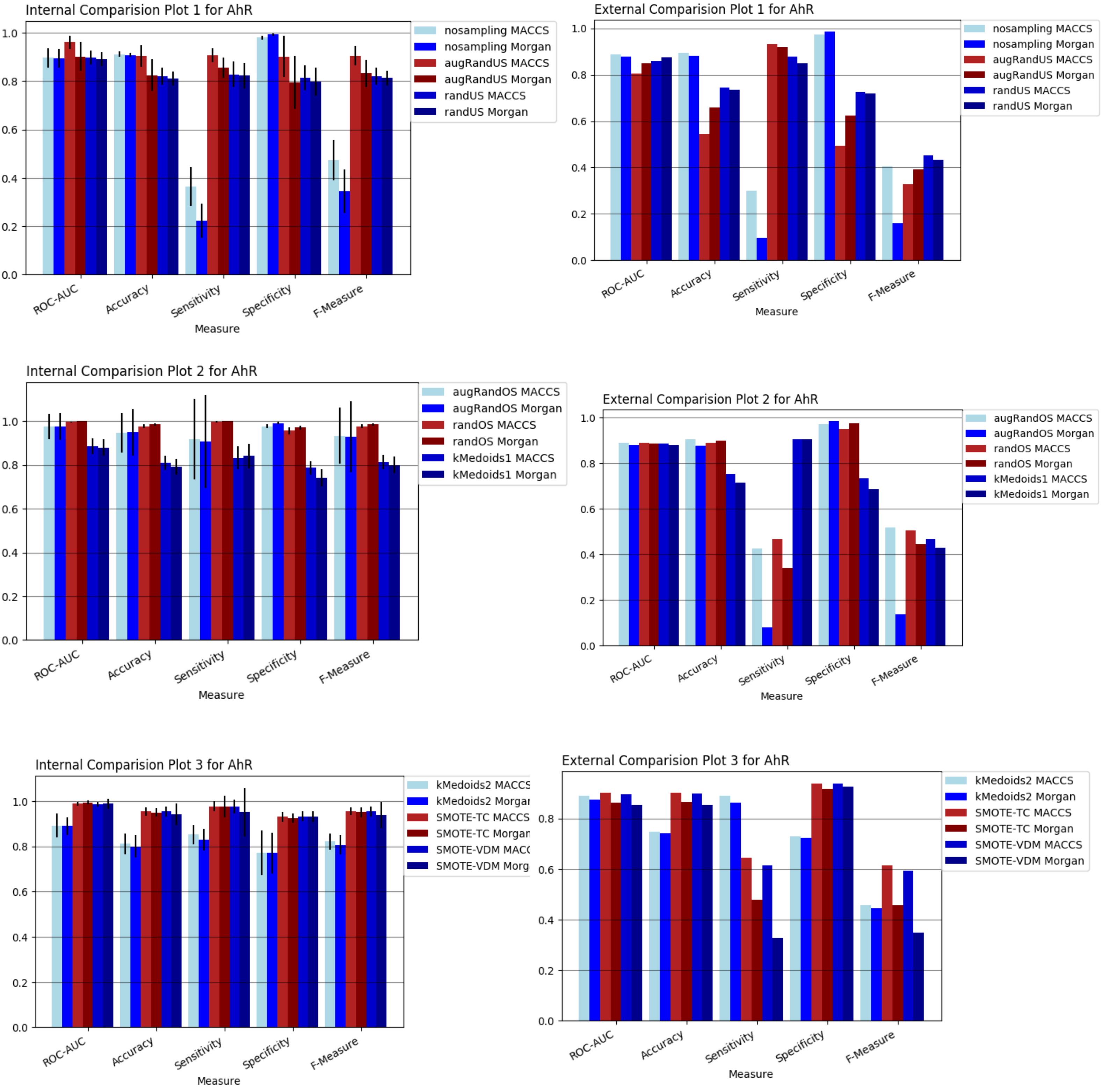

SIFigure9: Comparison between ER-LBD models based on MACCS and Morgan fingerprints with respect to of all performance measures for cross-validation (internal) and external validation.

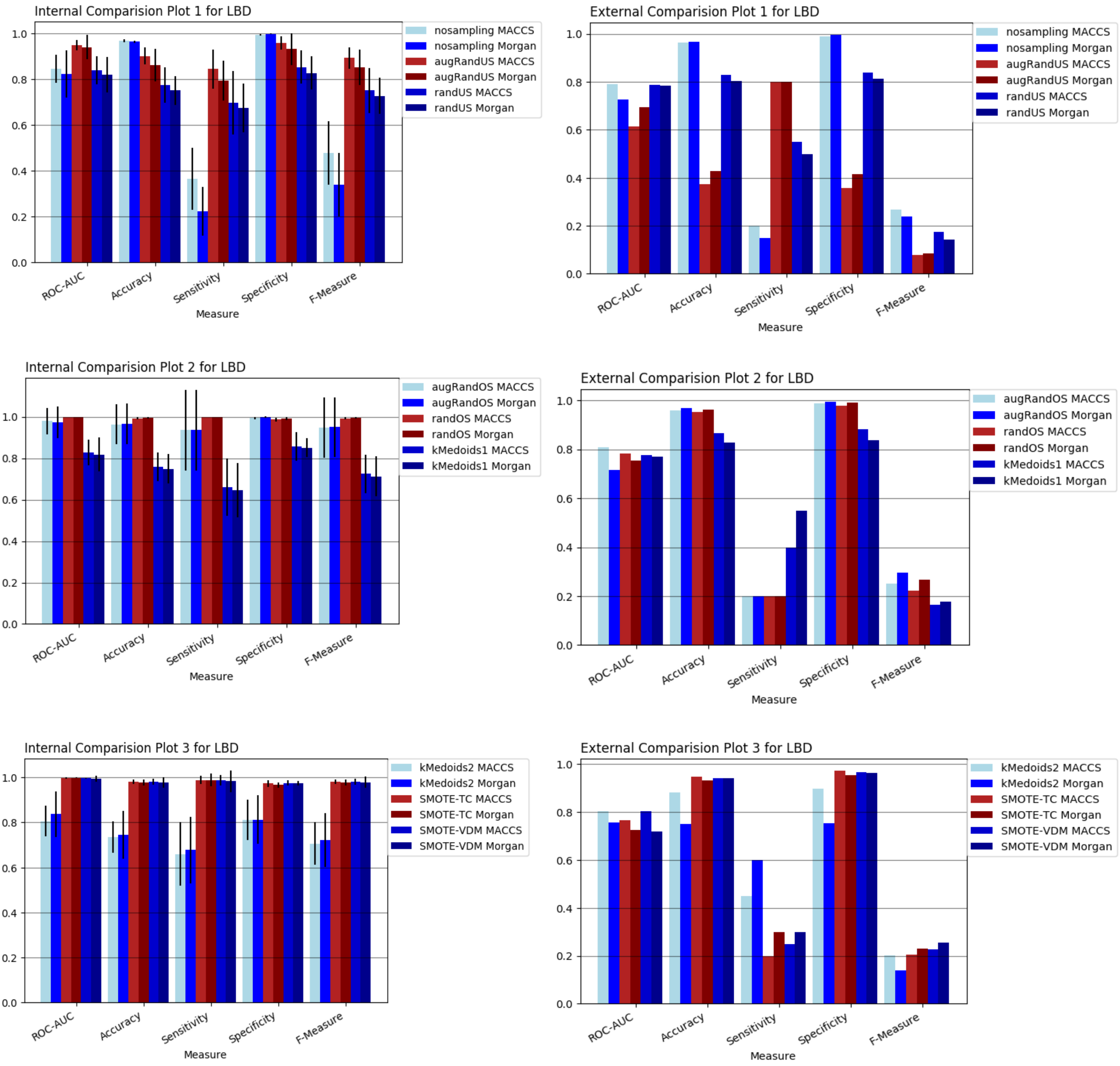

SIFigure10: Comparison between HSE models based on MACCS and Morgan fingerprints with respect to of all performance measures for cross-validation (internal) and external validation.

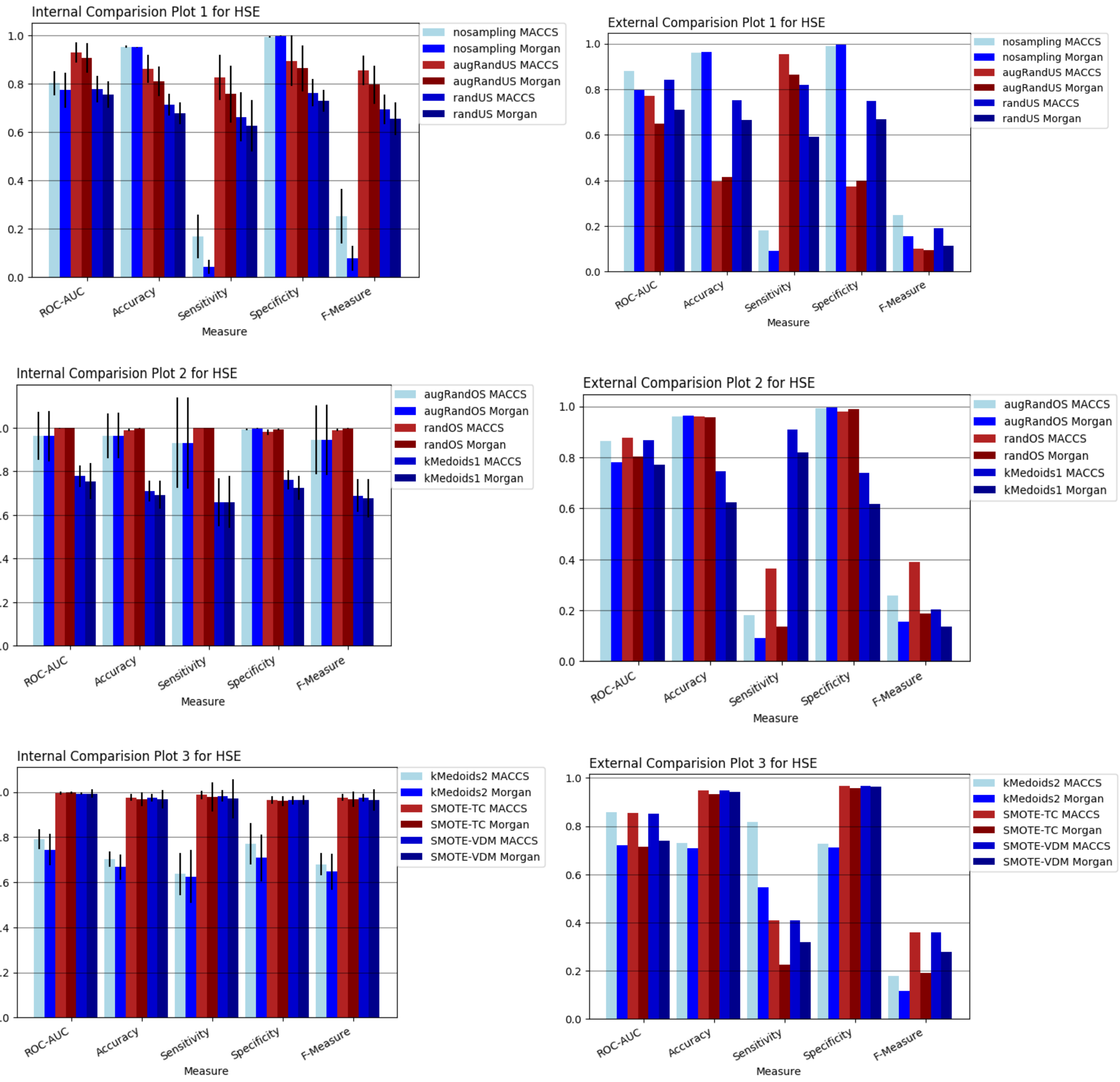

SIFigure11: Comparison of all the models based on MACCS and Morgan fingerprints with respect to of all precision measures for cross-validation (internal) and external validation.

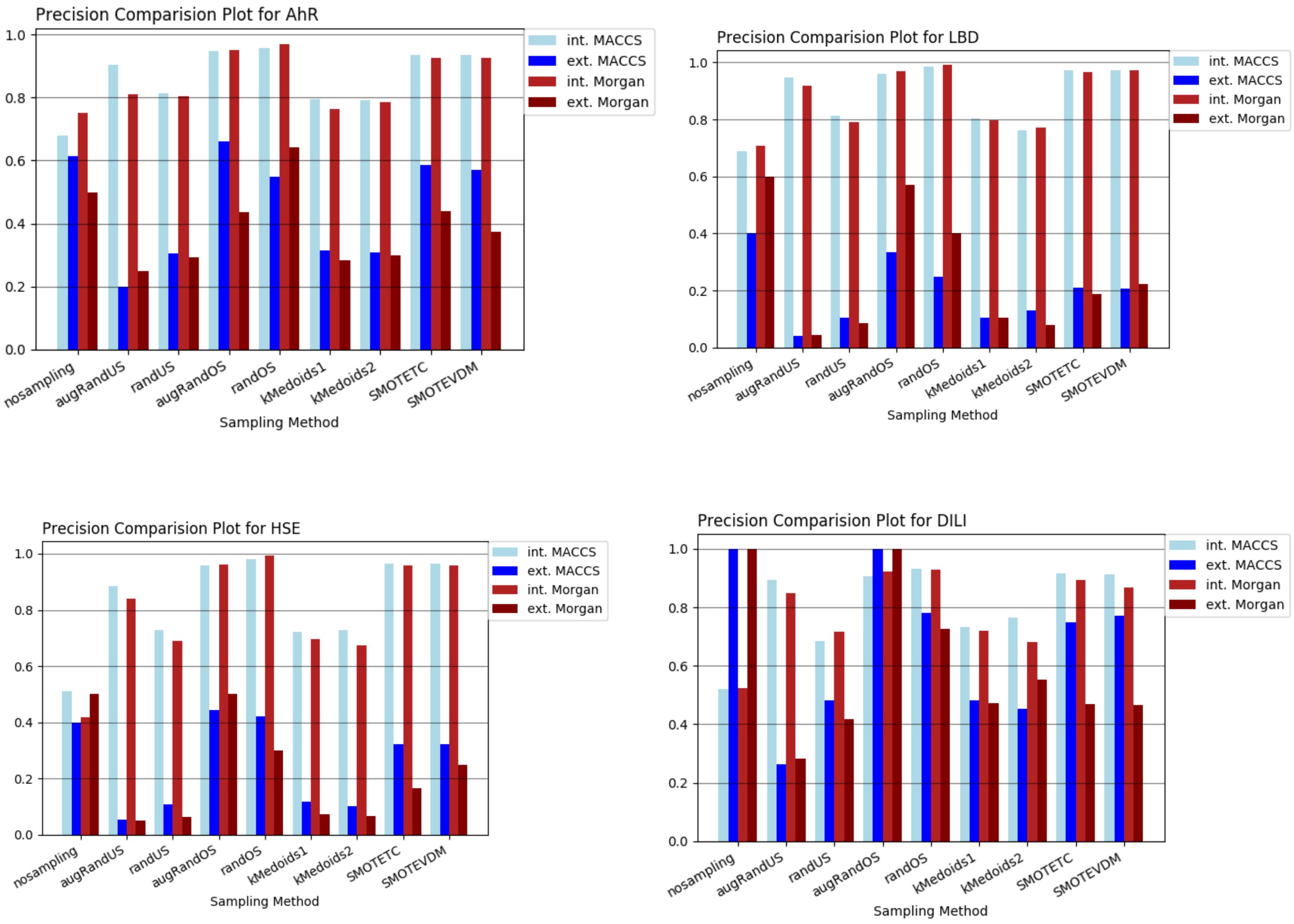

Supplement: Supplementary file 1 [file Data_Sheet_1.PDF]
